# Identification and Validation of Quantitative Trait Loci Mapping for Spike-Layer Uniformity in Wheat

**DOI:** 10.3390/ijms23031052

**Published:** 2022-01-19

**Authors:** Kunyu Zhou, Yu Lin, Xiaojun Jiang, Wanlin Zhou, Fangkun Wu, Caixia Li, Yuming Wei, Yaxi Liu

**Affiliations:** 1 State Key Laboratory of Crop Gene Exploration and Utilization in Southwest China, Chengdu 611130, China; zhoukunyu@stu.sicau.edu.cn (K.Z.); linyu@stu.sicau.edu.cn (Y.L.); jiangxj999@gmail.com (X.J.); zhouwanlin@stu.sicau.edu.cn (W.Z.); 2018112004@stu.sicau.edu.cn (F.W.); ymwei@sicau.edu.cn (Y.W.); 2 Triticeae Research Institute, Sichuan Agricultural University, Chengdu 611130, China; licaixia@sicau.edu.cn

**Keywords:** QTL mapping, spike-layer uniformity, kompetitive allele-specific PCR marker, yield traits, recombinant inbred lines, wheat

## Abstract

Spike-layer uniformity (SLU), the consistency of the spike distribution in the vertical space, is an important trait. It directly affects the yield potential and appearance. Revealing the genetic basis of SLU will provide new insights into wheat improvement. To map the SLU-related quantitative trait loci (QTL), 300 recombinant inbred lines (RILs) that were derived from a cross between H461 and Chinese Spring were used in this study. The RILs and parents were tested in fields from two continuous years from two different pilots. Phenotypic analysis showed that H461 was more consistent in the vertical spatial distribution of the spike layer than in Chinese Spring. Based on inclusive composite interval mapping, four QTL were identified for SLU. There were two major QTL on chromosomes 2BL and 2DL and two minor QTL on chromosomes 1BS and 2BL that were identified. The additive effects of *QSlu.sicau-1B*, *Qslu.sicau-2B-2,* and *QSlu.sicau-2D* were all from the parent, H461. The major QTL, *QSlu.sicau-2B-2* and *QSlu.sicau-2D*, were detected in each of the conducted trials. Based on the best linear unbiased prediction values, the two loci explained 23.97% and 15.98% of the phenotypic variation, respectively. Compared with previous studies, the two major loci were potentially novel and the two minor loci were overlapped. Based on the kompetitive allele-specific PCR (KASP) marker, the genetic effects for *QSlu.sicau-2B-2* were validated in an additional RIL population. The genetic effects ranged from 26.65% to 32.56%, with an average value of 30.40%. In addition, *QSlu.sicau-2B-2* showed a significant (*p <* 0.01) and positive influence on the spike length, spikelet number, and thousand kernel weight. The identified QTL and the developed KASP marker will be helpful for fine-mapping these loci, finally contributing to wheat breeding programs in a marker-assisted selection way.

## 1. Introduction

Wheat (*Triticum aestivum* L.) is one of the most important crops in the world, and it provides 20% of the global human population with calories [1,2]. To feed the world’s growing population, an annual rate increase of 2.4% in wheat productivity is expected. As the current annual rate of 0.9% is well below expectations, it is urgent to increase wheat productivity. Breeding high-yield wheat cultivars is one of the most effective strategies to increase the total food production.

Compared with conventional breeding methods, marker-assisted selection is an economic and efficient strategy to breed cultivars. Marker-assisted selection is a tool for breeders, allowing the possibility to select desirable traits more directly using DNA markers that are linked to genes/loci of interest [3]. Quantitative trait loci (QTL) mapping can provide usable molecular markers that are linked to a gene of interest. Up to now, QTL mapping has been widely applied to discover the locations of loci underlying traits in plants [4,5,6,7,8,9,10,11,12,13]. With the development of high-throughput sequencing technology, single nucleotide polymorphism (SNP) arrays, including the 9K, 55K, 90K, 660K, and 820K arrays, have been successfully used for constructing high-density genetic maps of wheat [14,15,16,17,18]. Based on this, QTL mapping has been used for detection within relatively narrow genetic intervals [14,15,19]. The availability of high-throughput SNPs and high-quality reference genomes combine to make the identification of QTL more precise. The identified QTL-linked markers, via dense genetic maps, could be directly used in wheat improvement through molecular assisted selection (MAS) and further accelerating fine mapping and map-based gene cloning.

Spike-layer uniformity (SLU), the consistency of the spike distribution in the vertical space, is determined by the different plant heights (PH) between the main stem and tillers. A uniform spike-layer ensures, not only uniform grain sizes, but also a synchronized maturation time and facilitates harvesting [20]. In rice, ideotype plants with a high yield potential are suggested to have uniformity in the panicle layer [20]. Several genes have been demonstrated to control SLU and affect yield [21,22]. In wheat, the SLU is a quantitative trait and directly affects the yield potential and appearance [23,24]. It has relatively high heritability and is affected by both genotypes and environmental factors [23,24]. A previous study found that the genotype “Jing411” has a higher SLU value than “Kenong9204” [23]. This indicated that “Jing411” has a more consistent vertical spatial distribution. To identify the wheat genotypes with uniform spike-layers and loci that are associated with this trait, a natural population, including 225 diverse bread wheats, was also used in a previous study. In previous studies, QTL for SLU have been identified on chromosomes 1A, 1B, 1D, 2A, 2B, 2D, 3A, 3B, 4A, 4B, 5A, 5B, 6A, 6B, 6D, 7A, and 7D [23,24]. Among these, only one locus, *qSlu-4A*, was detected in three environments; the others were identified in one or two environments. To breed wheat with consistent spike heights, it is necessary to detect stable QTL of SLU among multiple environments. These expected QTL were reliable in the marker-assisted selection of wheat improvement.

Spike-layer uniformity is an important trait that directly affects wheat yield potential and appearance. Uncovering the genetic basis of SLU in wheat will provide a new insight into wheat improvement. In this study, the SLU of H461 was more consistent than that of Chinese Spring. The derived recombinant inbred line (RIL) population was used to evaluate the SLU from two consecutive years from two different environments. Based on the high-density map that was constructed by the 55K SNP array, QTL mapping was performed for this trait in multiple environments and using the best linear unbiased prediction (BLUP) values. The genetic effects of the objective locus were validated in a different background using an additional RIL population. Further analysis was performed to reveal the genetic relationships between the SLU and several yield traits.

## 2. Results

### 2.1. Phenotypic Characterization of Spike-Layer Uniformity

**Figure 1:**
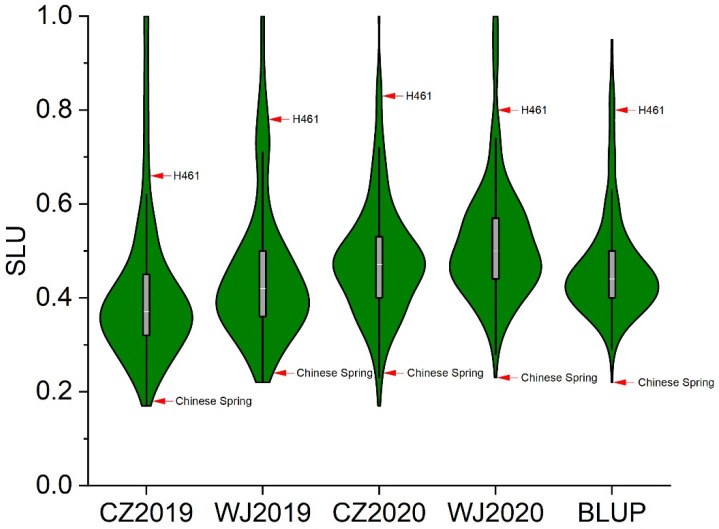
Violin plots for spike-layer uniformity distributions of the H461 × Chinese Spring recombinant inbred line population in different environments. CZ2019, CZ2020, WJ2019, and WJ2020 denote field experiments in Chongzhou (2019, 2020) and Wenjiang (2019, 2020). BLUP, best linear unbiased prediction.

A significant (*p* < 0.01) difference was identified for the SLU among genotypes, environments, and genotype–environment interactions (Table 1). In all four trials, the SLU of H461 ranged from 0.66 to 0.83, while the SLU of Chinese Spring ranged from 0.18 to 0.24 (Figure 1, Table 2). The spike-layer uniformity of H461 was significantly higher than that of Chinese Spring in all four trials (Table 2). Based on the BLUP values, the SLU of H461 was 0.80, which was 3.6 times higher than that of Chinese Spring. The results revealed the great potential of wheat spike-layer improvement using the material H461. In all the trials, transgressive segregation was observed in the RIL population (Figure 1, Table 2). The broad-sense heritability of SLU was 0.88, indicating that this trait was majorly controlled by genetic factors. Significant (*p* < 0.01) correlations were observed among all four environments with correlation coefficients ranging from 0.62 to 0.79 (Supplementary Table S1), which indicated that the SLUs that were obtained from the four trials were strongly correlated.

### 2.2. Identification of QTL Conferring Spike-Layer Uniformity

**Figure 2:**
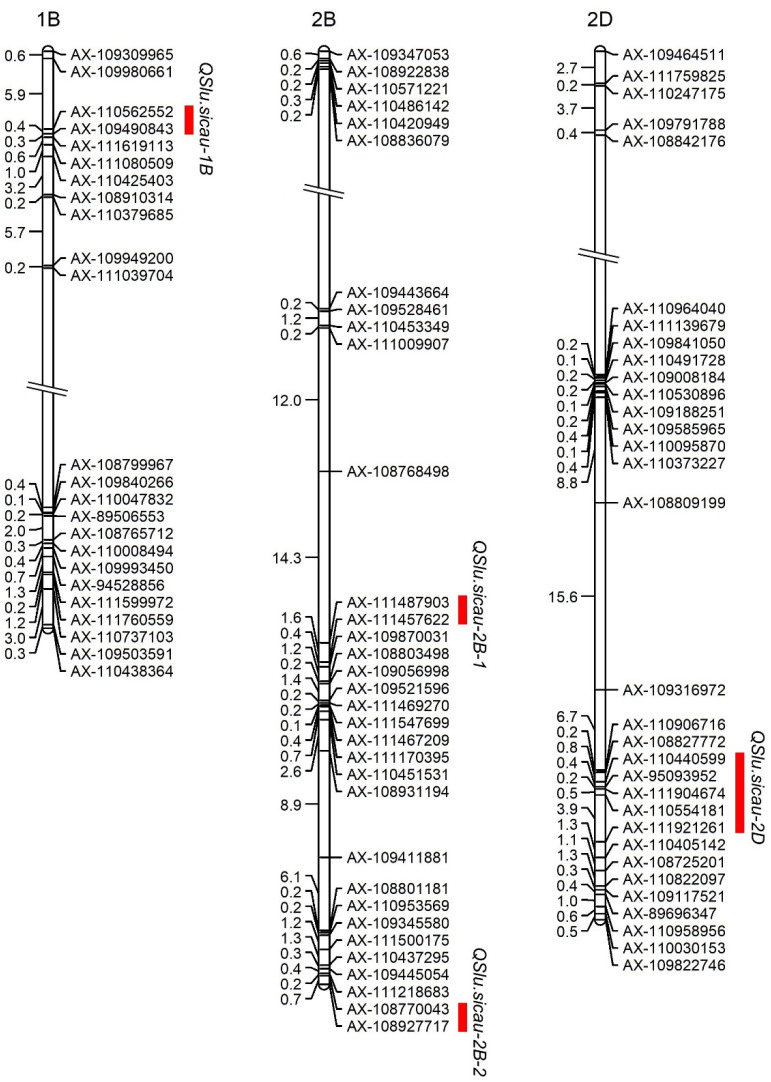
Chromosomal locations of QTL for spike-layer uniformity in the H461 × Chinese Spring recombinant inbred line population. The red bar points to the genetic interval of QTL. The numbers on the left side of the chromosomes point to the genetic distance (cM) between the markers. The marker’s names are shown on the right side of chromosomes.

A total of four QTL were identified for the SLU (Figure 2, Table 3). Among them, two major QTL were located on chromosomes 2B (*QSlu.sicau-2B-2*) and 2D (*QSlu.sicau-2D*), and two minor QTL were located on chromosomes 1B (*QSlu.sicau-1B*) and 2B (*QSlu.sicau-2B-1*). *QSlu.sicau-2B-2* (flanked by AX-108770043 and AX-108927717) was detected in all four trials, as well as in the BLUP values. This locus was located on the long arm of chromosome 2B at 165.70–166.40 cM and explained 13.89–23.97% of the phenotypic variation. The other major locus, *QSlu.sicau-2D,* was detected in all the trials, as well in the BLUP values. It was located on the long arm of chromosome 2D at 230.67–235.60 cM and explained 12.93–15.98% of the phenotypic variation. The minor locus *QSlu.sicau-1B* (flanked by AX-109490843 and AX-111619113) was detected in CZ2020, as well as in the BLUP values and was located on the short arm of chromosome 1B at 6.87–7.22 cM, explaining 3.43–3.55% of the phenotypic variation. The other minor locus, *QSlu.sicau-2B-1* (flanked by AX-111487903 and AX-111457622), was detected in WJ2019 as well as in the BLUP values, explaining 2.05–3.91% of the phenotypic variation. This locus was on the long arm of chromosome 2B at 137.91–139.55 cM. With the exception of *QSlu.sicau-2B-1*, alleles for the higher SLUs at these loci were all from the parent, H461.

### 2.3. Validation of the Major and Stable QTL, with a Different Genetic Background and Its Effect on Yield-Related Traits

**Figure 3:**
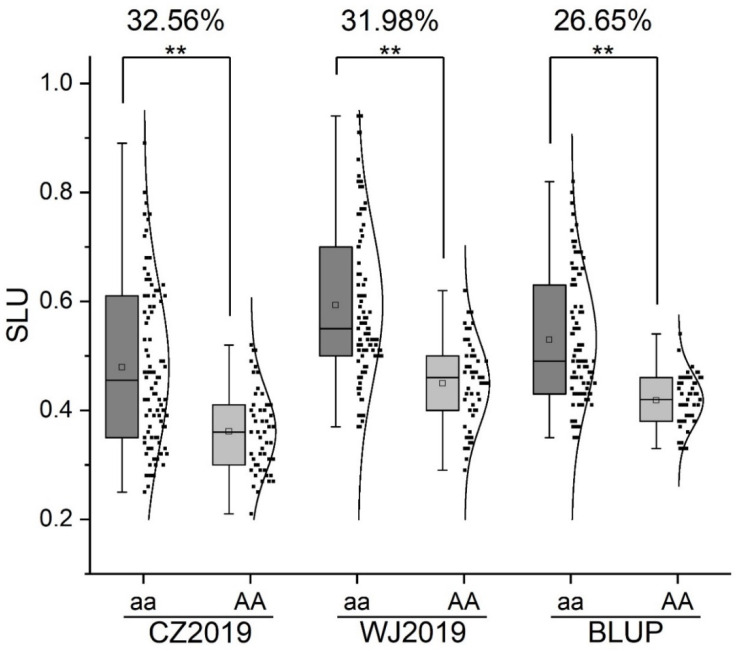
Effects of *Qslu.sicau-2B-2* in the H461 × CM42 recombinant inbred line population. Aa, lines carrying the homozygous alleles from H461. AA, lines carrying the homozygous alleles from the non-H461 parents. CZ2019 and WJ2019 denote the field experiments in 2019 in Chongzhou and Wenjiang, respectively. BLUP, best linear unbiased prediction. **, significant at *p* < 0.01.

The tightly linked SNP, AX-108927717, was converted into a kompetitive allele-specific PCR (KASP) marker (KASP-7717) to validate the major and stable locus, *QSlu.sicau-2B-2*, with a different genetic background (Table 4). The KASP marker was polymorphic, between H461 and the other two parents (Chinese Spring and CM42). In the RILs that were derived from H461 and CM42, the SLU of the group carrying the “aa” allele was significantly (*p* < 0.01) higher than that of the group carrying the “AA” allele (Figure 3). The differences between the two groups were 32.56%, 31.98%, and 26.65% for CZ2019, WJ2019, and BLUP, respectively, with an average value of 30.40% (Figure 3). In addition, the spike length (SL) of the group carrying the “aa” allele was significantly (*p* < 0.05) higher than that of the group carrying the “AA” allele. The group carrying the “aa” allele had a significantly (*p* < 0.01) higher spikelet number (SN) and thousand kernel weight (TKW) than the group carrying the “AA” allele. Thus, *QSlu.sicau-2B-2* showed a significant and positive influence on SL, SN, and TKW, but it did not affect PH (Table 5). These results indicated that this locus has great potential in wheat yield improvement.

### 2.4. Candidate Genes for QSlu.sicau-2B-2

Based on the Chinese Spring Reference Genome Sequence RefSeq v2.1 [25,26], *QSlu.sicau-2B-2* was located on the long arm of chromosome 2B at 798.21–802.29 Mb. A total of 49 predicted genes were identified in the interval. After homologous analysis, a total of 47 genes were homologous with rice. There were two homologous genes of rice that have been reported regarding their functions, *GSTU5* [27] and *GSTU45* [27]. Expression analysis of these genes is shown in Figure S1. *TraesCS2B03G1529700*, which encodes thioredoxin, showed the highest expression, from the seedling stage to the dough stage (Table S2). The expressions of eight genes, including *TraesCS2B03G1522100*, *TraesCS2B03G1522700*, *TraesCS2B03G1524300*, *TraesCS2B03G1526400*, *TraesCS2B03G1531500*, *TraesCS2B03G1531600*, and *TraesCS2B03G1534400*, were shown to be highly expressed in all developmental stages.

## 3. Discussion

The uniformity of the spike layer significantly affects the plant architecture and grain yield in wheat. The high heritability of SLU in the present study indicated that genetic factors strongly determined this trait (Table 1). The high heritability for SLU was also observed in the population that included 20 wheat cultivars/lines [28]. Previous studies also confirmed that genetic factors mainly controlled SLU [22,28,29]. Thus, it is necessary to understand the genetic basis of SLU for MAS to be applied in wheat breeding.

The genetic basis of SLU is poorly understood in wheat. In rice, a few genes have been demonstrated to control plant uniformity, including *DWT1*, *DWL2,* and *OsPIP5K1* [21,22]. *DWT1* encodes a *WOX* transcription factor and interacts with *OsPIP5K1* to coordinate rice uniformity [21,22]. *DWL2* has the same redundant functions as *DWT1* and acts with *OsPIP5K1* to coordinate uniformity in rice [21]. To the best of our knowledge, only two studies on QTL identification for SLU have been reported [23,24]. A total of 19 QTL have been detected and are located on chromosomes 1B, 1D, 2A, 2B, 2D, 4A, 4B, 5A, 5B, 6B, 7A, and 7D. Based on the RIL population consisting of 188 lines, 16 QTL were detected on chromosomes 1B, 1D, 2A (2 QTL), 2B (2 QTL), 4A, 4B, 5A, 5B, 6B (2 QTL), 7A (2 QTL), and 7D (2 QTL), explaining 5.04–18.43% phenotypic variation. Of those, one (*qSlu-4A*) could be detected across three environments and two (*qSlu-4B* and *qSlu-6B.1*) across two environments [23]. In the present study, only four QTL were detected for SLU (Figure 2, Table 3). This is due to the loci that were detected in only one environment being removed in this study. It is well known that QTL that are detected in one environment are not useful in breeding. Due to the significant differences between parents, the large population, and abundant polymorphism markers, two stable and major QTL were detected in this study. Recently, using a set of 225 diverse spring wheat accessions, 14 marker-trait associations for SLU were detected on chromosomes 1A, 1B, 2D, 3A, 3B, 4A, 4B, 5A, 5B, 6A, 6B, 6D, and 7A [24]. Only the SNP, SNP_10924, was significantly associated with the SLU in two environments, explaining up to 6% of the phenotypic variation [24]. These results indicated that detecting a more stable and major QTL for SLU is necessary.

Based on the Chinese Spring Reference Sequence RefSeq v2.1 [25,26], QTL were compared with previous studies [23,24]. In this study, one minor-effect QTL, *QSlu.sicau-1B*, was identified on chromosome 1B at 572.94–577.58 Mb. In a previous study, the marker SNP_11184 was located on the short arm of chromosome 1B and was significantly associated with SLU [24]; it was far from *QSlu.sicau-1B*. In another study, the locus *qSlu-1BL* was identified on chromosome 1B in the interval between wPt-2315 and AX-108850061 [23]. Due to the unavailable physical position of the DArT marker wPt-2315, the physical position of this marker was referred to by the adjacent marker, AX-111595814. Thus, *qSlu-1BL* is probably located on chromosome 1B at 575.35–587.26 Mb, overlapping with *QSlu.sicau-1B*. *QSlu.sicau-2B-1* was another minor-effect QTL. This QTL was located on chromosome 2B at 755.21–777.78 Mb, overlapping with *qSlu-2B.2* (flanked by AX-111041164 and AX-94403958) [23]. The locus *qSlu-2B.2* was located on chromosome 2B at 735.06–779.53 Mb. *QSlu.sicau-2B-2* was a major and stable QTL with the highest effect. This QTL was located on the long arm of chromosome 2B at 798.21–802.29 Mb. It is far from *qSlu-2B.1* (chromosome 2B at 725.48-725.76 Mb) and *qSlu-2B.2* (chromosome 2B at 735.06-779.53 Mb), indicating that *QSlu.sicau-2B-2* is a potential novel QTL for SLU. *QSlu.sicau-2D* is another QTL with a major effect. This QTL is located on the long arm of chromosome 2D at 565.41–640.84 Mb. One of the SLU-related QTL on the short arm of chromosome 2D was reported previously [24], indicating *QSlu.sicau-2D* as another potential novel QTL. A tightly linked marker of *QSlu.sicau-2B-2* was designed to be a KASP marker that was applied in wheat molecular breeding programs (Table 4). The KASP marker, KASP-7717, was genotyped in the HC42 population. The locus *QSlu.sicau-2B-2* showed a great effect in a different background with an average difference of 30.40% (Figure 3). This result indicated that *QSlu.sicau-2B-2* in MAS for plant architecture improvement in wheat is practicable.

Spike-layer uniformity is a vital plant architecture trait and affects the yield potential in crops with tillers, such as rice, barley, and wheat. Previous studies have found that the SLU and yield potential were positively correlated [22,29,30,31]. It has been demonstrated that DWT1 regulates intra-panicle uniformity and coordinates panicle and internodes in rice [22]. In wheat, knowledge of the genetic relationships between the SLU and yield-related traits is limited. A previous study found that the SLU was significantly negatively correlated with PH, TKW, and the yield per plant [23]. In the present study, *QSlu.sicau-2B-1*, located on chromosome 2B at 755.21–777.78 Mb, was overlapped with the interval of *QSl.sicau-2B-1* (Table 3; Table S3). The additive effects of these two QTL were both from Chinese Spring. This indicated that this locus could regulate both the SLU and SL and not the lowest tiller height (LTH) or PH. *QSlu.sicau-2B-2*, located on chromosome 2B at 798.21–802.29 Mb, was overlapped with the interval of *QGns.sicau-2B* [14]. This locus was also overlapped with the interval of *QSl.sicau-2B-2* (Table 3, Table S3). The additive effects of these three QTL were both from H461. This indicated that this locus could regulate SLU and coordinate the grain number per spikelet and SL. In the RIL population that was derived from the crossing of H461 and CM42, *QSlu.sicau-2B-2* also had a positive influence on SL, SN, and TKW, but did not affect PH (Table 5). This finding was inconsistent with previous results [23] indicating that the mechanism of *QSlu.sicau-2B-2* may be different. This locus could improve the uniformity of the spike layer and several yield-related traits, demonstrating that applications of *QSlu.sicau-2B-2* in wheat yield improvement by MAS are feasible and necessary. A total of 49 genes were located in the interval of *QSlu.sicau-2B-2*. Of those, eight showed high expression in the wheat development stages (from the seedling stage to the dough stage, Figure S1 and Table S2). *TraesCS2B03G1529700* encodes chaperone protein DnaJ (Table S2). A previous study also found that the gene that encodes chaperone protein DnaJ was associated with wheat spike development, seed development, and the grain yield [24]. Combined with expression analyses, *TraesCS2B03G1529700* may be the most critical candidate gene for this locus. To validate it, qRT-PCR and transgene testing should be conducted in further studies.

## 4. Materials and Methods

### 4.1. Plant Materials, Trial Design, and Phenotype Evaluation

A total of 300 RILs (F_8_) that were derived from the cross between H461, and Chinese Spring (denoted as the HCS population) were used to map the SLU-related QTL in this study [14]. In addition, 200 RILs (F_8_) that were derived from the crossing of H461 and CM42 (denoted as the HC42 population) were used to validate the genetic effects of the major QTL. The SLU of H461 was significantly higher than that of Chinese Spring and CM42.

The HCS population was evaluated in Chongzhou (30°32′ N, 103°38′ E) in 2019 (CZ2019) and 2020 (CZ2020), and in Wenjiang (30°43′ N, 103°51′ E) in 2019 (WJ2019) and 2020 (WJ2020). The HC42 population was evaluated in CZ2019 and WJ2019. Chongzhou and Wenjiang have yellow–brown soil and paddy soil, respectively. The plant dates and meteorological data, including the temperature and rainfall, of these four trials are presented in Table S4. The experiment was set as an incomplete block design with two repetitions for each environment. Each line contained 15 plants that were grown in a single row for each repetition. The row length was 150 cm and the row spacing was 30 cm. Standard irrigations, fertilization management, and pest and disease preventions were performed throughout the developmental period. The lowest tiller height (LTH), PH, and SL of the HCS population were evaluated in all four environments. The LTH, PH, SL, SN, and TKW of the HC42 population were also assessed. Each plant’s lowest and highest productive tiller was used to evaluate the LTH and PH, respectively. The LTH and PH were measured from the ground level to the tip of the spike, excluding awns. The SL was measured from the base to the tip of the spike, excluding awns. The SN was counted as the number of fertile spikelets per spike. TKW was measured as twice the weight of 500 kernels, harvested in the middle row at the maturation stage. The SLU was calculated as SL/(PH-LTH+SL), as shown in Figure S2.

### 4.2. Phenotypic Data Analysis

The average values of each conducted trial were calculated using SPSS 20.0 (IBM Corp., Armonk, NY, USA). The BLUP values were calculated across all the conducted trials using SAS 9.4 (SAS Institute Inc., Cary, NC, USA), as reported previously [32,33]. The broad-sense heritability of SLU was calculated as *H*^2^ = Vg/(Vg + Vge/n + Ve/nr); where, Vg, Vge, and Vg are the estimates of variances of genotype, genotype × environment interaction, and random error variances, respectively, while n and r are the number of environments and replicates [34]. The Student’s *t*-test was used to evaluate the SLU differences between H461 and Chinese Spring.

### 4.3. Genotyping, Map Construction, QTL Mapping

The HCS population was genotyped using the wheat 55K SNP array containing 53,063 markers from our previous study [14]. The high-density linkage map of the HCS population contained 21,197 SNPs (represented by 3087 bin markers), spanning 3792.71 cM in total, as described in our previous study [14]. The linkage map contained 21 groups corresponding to 21 wheat chromosomes, respectively. QTL mapping for SLU was detected using the inclusive composite interval mapping [35] in IciMapping 4.2 [36]. To increase the authenticity and reliability of the QTL detected, the threshold was set as LOD scores ≥3, and the loci that were detected in only one environment or only using BLUP values were removed [37,38,39]. QTL were named according to the rules that were established by the International Rules of Genetic Nomenclature (http://wheat.pw.usda.gov/ggpages/wgc/98/Intro.htm (accessed on 12 September 2021)). For example, “Slu” and “sicau” represent “spike-layer uniformity” and “Sichuan Agricultural University,” respectively. The flanking markers of the QTL were mapped on the Chinese Spring Reference Sequence RefSeq v2.1 to obtain their chromosomal and physical locations [25,26].

### 4.4. Marker Development, QTL Validation, and Candidate Genes Prediction

SNP probe sequences flanking the target QTL were used to convert them into KASP markers. KASP primers were designed and analyzed following the procedure that was described by Suzuki et al. (1991), as in our previous report [40,41]. The amplification reactions were performed in a total volume of 10 μL, containing 5 μL of 2 × KASP Mastermix (JasonGen, Beijing, China), 1.4 μL of KASP Assay Mix (mixture of 0.168 μmol of FAM primer, 0.168 μmol of HEX primer, and 0.42 μmol common reverse primer), 2.6 μL of DNase/RNase-free water, and 1 μL of genomic DNA (concentration of ~100 ng/μL). The amplifications were carried out using the CFX96Touch^TM^ real-time PCR detection system (BioRad, Hercules, California, USA) with the following steps: 95 °C for 15 min, 10 touchdown cycles for 65–55 °C (decreasing by 1 °C per cycle) for 60 s, and 30 cycles for 94 °C for 20 s and 57 °C for 60 s.

For the HCM population, 141 randomly selected lines were genotyped using the designed KASP marker. The RILs were classed into two groups based on the alleles that were present in the parents and their lines. The lines carrying homozygous alleles from H461 and non-H461 parents were denoted as “aa” and “AA”, respectively. The Student’s *t*-test was used to calculate the significances of the traits between the two groups. Based on Chinese Spring Reference Sequence RefSeq v2.1, the predicted genes that were located in the physical interval of the QTL were determined using homologous analysis by KOBAS 3.0 [42] and funRiceGenes [43] using rice (*Oryza sativa*) as the background species. In silico gene expression analysis was also performed to identify candidate genes from the Wheat Expression Browser (http://www.wheat-expression.com/ (accessed on 7 January 2022)).

## 5. Conclusions

The wheat line H461 was more consistent in the vertical spatial distribution of the spike layer than Chinese Spring. The spike-layer uniformity of 300 RILs that were derived from these two parents was evaluated in multiple environments. Combined with a previously constructed high-density genetic map, two major-effect QTL were successfully identified on the long arms of chromosomes 2B and 2D, respectively. There were two minor-effect QTL that were identified on the short arm of chromosome 1B and the long arm of chromosome 2B. A comparison analysis that was based on a physical map of Chinese Spring indicated that the two major loci, *QSlu.sicau-2B-2* and *QSlu.sicau-2D*, were probably novel. The effect of *QSlu.sicau-2B-2* was validated in an additional RIL population using a developed KASP marker. *QSlu.sicau-2B-2* also positively affected SL, SN, and TKW. The results indicated that this QTL and KASP marker could be used in MAS for new wheat cultivar breeding with a scientifically reasonable spike-layer distribution.

## Figures and Tables

**Table 1:** Analysis of variance for spike-layer uniformity across the four environments.

Variable	DF ^a^	Type III Sum of Square	Mean Square	F Value	Significance ^b^
Environment	3	3.76	1.25	141.77	**
Replication	4	0.02	0.005	0.52	ns
Genotype	298	32.51	0.11	12.35	**
Environment × Genotype	885	10.29	0.01	1.32	**

^a^ DF, degrees of freedom. ^b^ **, significant at *p* < 0.01; ns, not significant.

**Table 2:** Phenotypic variation and the broad-sense heritability of spike-layer uniformity in different environments.

Environment ^a^	Parents			Recombinant Inbred Line (RIL) Population					
	H461	Chinese Spring	Difference ^b^	Mean	Min	Max	SD ^c^	CV (%) ^d^	Heritability
CZ2019	0.66	0.18	0.49 **	0.41	0.17	1.00	0.15	37.61	
WJ2019	0.78	0.24	0.53 **	0.45	0.22	1.00	0.15	32.84	
CZ2020	0.83	0.24	0.59 **	0.48	0.17	1.00	0.12	25.11	
WJ2020	0.80	0.23	0.57 **	0.52	0.28	1.00	0.13	24.34	
BLUP	0.80	0.22	0.58 **	0.58	0.29	0.95	0.11	18.69	0.88

^a^ CZ2019, CZ2020, WJ2019, and WJ2020 denote field experiments in Chongzhou (2019, 2020) and Wenjiang (2019, 2020). BLUP, best linear unbiased prediction. ^b^ **, significant at *p* < 0.01. ^c^ SD, standard deviation. ^d^ CV, coefficient of variation.

**Table 3:** Quantitative trait loci for spike-layer uniformity that were identified in different environments and using BLUP values.

QTL	Environment ^a^	Chromosome	Genetic Distance (cM)	Physical Positions (Mb)	Flanking Marker	LOD ^b^	PVE (%) ^c^	AE ^d^
*QSlu.sicau-1B*	CZ2020	1B	6.87–7.22	572.94–577.58	AX-109490843 and AX-111619113	3.73	3.43	0.024
	BLUP	1B	6.87–7.22	572.94–577.58	AX-109490843 and AX-111619113	5.96	3.55	0.024
*QSlu.sicau-2B-1*	WJ2019	2B	137.91–139.55	755.21–777.78	AX-111487903 and AX-111457622	6.17	3.91	−0.034
	BLUP	2B	137.91–139.55	755.21–777.78	AX-111487903 and AX-111457622	3.47	2.05	−0.018
*QSlu.sicau-2B-2*	CZ2019	2B	165.70–166.40	798.21–802.29	AX-108770043 and AX-108927717	17.66	17.98	0.070
	WJ2019	2B	165.70–166.40	788.13–792.19	AX-108770043 and AX-108927717	28.76	21.21	0.078
	CZ2020	2B	165.70–166.40	788.13–792.20	AX-108770043 and AX-108927717	14.06	13.89	0.048
	WJ2020	2B	165.70–166.40	788.13–792.21	AX-108770043 and AX-108927717	16.12	16.73	0.055
	BLUP	2B	165.70–166.40	788.13–792.22	AX-108770043 and AX-108927717	32.70	23.97	0.062
*QSlu.sicau-2D*	CZ2019	2D	231.72–235.60	565.41–640.84	AX-110554181 and AX-111921261	13.61	13.67	0.062
	WJ2019	2D	230.67–231.02	633.66–634.41	AX-110440599 and AX-95093952	18.98	12.93	0.061
	CZ2020	2D	231.72–235.60	565.41–640.84	AX-110554181 and AX-111921261	14.46	14.88	0.050
	WJ2020	2D	231.72–235.60	565.41–640.84	AX-110554181 and AX-111921261	14.84	15.39	0.053
	BLUP	2D	230.67–231.02	633.66–634.41	AX-110440599 and AX-95093952	23.75	15.98	0.051

^a^ CZ2019, CZ2020, WJ2019, and WJ2020 denote field experiments in Chongzhou (2019, 2020) and Wenjiang (2019, 2020). BLUP, best linear unbiased prediction. ^b^ LOD, the logarithm of odds score. ^c^ PVE, the percentage of phenotypic variance explained by individual QTL. ^d^ AE, additive effect. Positive values mean alleles from H461 increase the trait scores, and negative values mean alleles from Chinese Spring increase the trait scores.

**Table 4:** The primers of the kompetitive allele-specific marker for alleles of *Qslu.sicau-2B-2* that were used in this study.

SNP	Primer ^a^	Primer sequence (5’ to 3’)	Allele ^b^
AX-108927717	FAM	TTGGAATGTCTCCATCCCAC	aa
	HEX	TTGGAATGTCTCCATCCCAG	AA
	Common reverse	CCTCTCCTATATCTGGCTTCTGTTG	

**^a^** FAM probe sequence of the forward primer is GAAGGTGACCAAGTTCATGCT, and the HEX probe sequence of the reverse primer is GAAGGTCGGAGTCAACGGATT. ^**b**^ aa, lines carrying the homozygous alleles from H461; AA, lines carrying the homozygous alleles from the non-H461 parents.

**Table 5:** Effects of *QSlu.sicau-2B-2* on the yield-related traits in the H461 × HC42 recombinant inbred line population.

Environment ^a^	Allele	Plant Height (cm)	Spike Length (cm) ^b^	Spikelet Number	Thousand Kernel Weight (g)
CZ2019	aa	84.22	13.60 *	22.11 **	50.15 **
	AA	84.22	13.23	20.81	45.84
WJ2019	aa	82.01	13.45 **	20.92 **	50.15 **
	AA	82.01	12.80	19.74	45.84
BLUP	aa	83.10	13.50 *	21.48 **	47.58 **
	AA	81.25	13.05	20.35	44.60

^a^ CZ2019 and WJ2019 denote field experiments in 2019 in Chongzhou and Wenjiang, respectively. BLUP, best linear unbiased prediction. ^b^ *, significant at *p* < 0.05. **, significant at *p* < 0.01.

## Data Availability

The data presented in this study are available on request from the corresponding author.

## Supplementary Materials

The following are available online at https://www.mdpi.com/article/10.3390/ijms23031052/s1.
