# Supplementary material for: Identification and Validation of Quantitative Trait Loci Mapping for Spike-Layer Uniformity in Wheat
Source: Int J Mol Sci. 2022 Jan 19;23(3):1052. doi: 10.3390/ijms23031052 (PMC8835109; doi:10.3390/ijms23031052)
Supplement: Supplementary file 1 [file ijms-23-01052-s001.zip › Supplementary Figures.pdf]

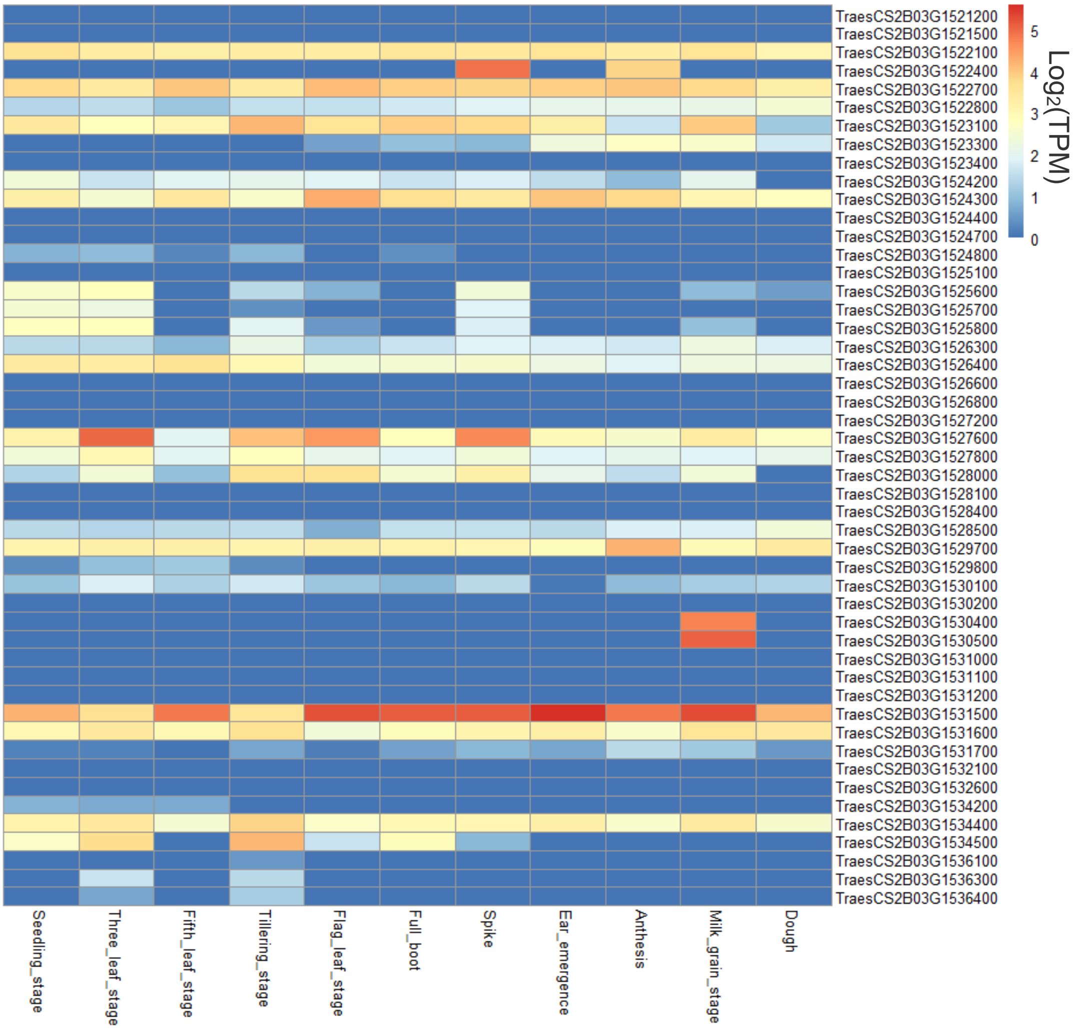

Supplementary Figure S1 Heat map of in silico gene expression analysis for predicted genes in the interval of *Qslu.sicau-2B-2*. The RNA-seq expression data of these predicted genes are from the wheat expression browser (http://www.wheat-expression.com/).

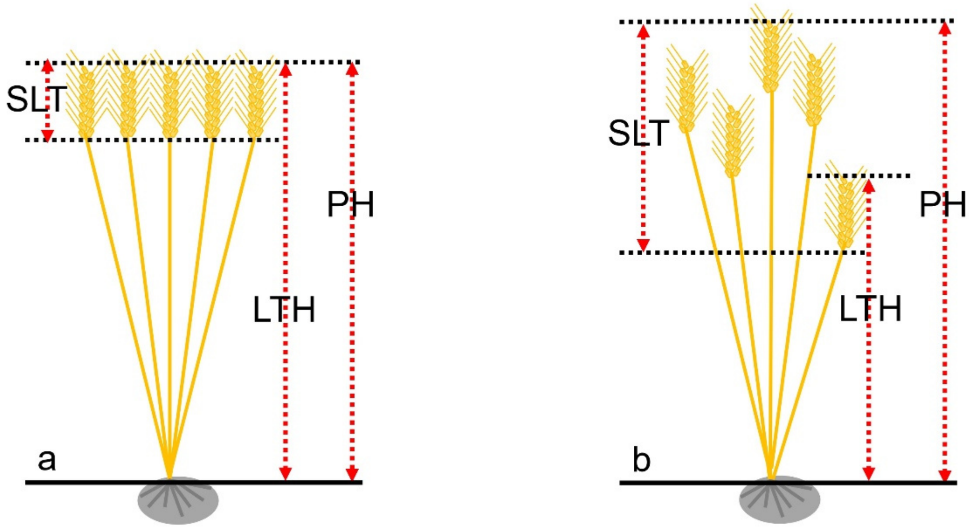

Supplementary Figure S2 The intuitional visualization of the phenotype was investigated in this study. Spike-layer thickness (SLT) was calculated as PH-LTH+SL. Each line's spike-layer uniformity (SLU) was calculated as SL/(PH-LTH+SL). **(a)** The plant sample shows that the most consistent spikes are distributed in the vertical space (SLU=1). All tillers have the same height, indicating that LTH equals PH. **(b)** The plant sample shows inconsistent spikes are distributed in the vertical space (0<SLU<1). Note: LTH, lowest tiller height; PH, plant height, SL, spike length.
